# Commentary: Case Report: CAR-T cell therapy bridging to allogeneic hematopoietic stem cell transplantation triggers Purtscher-like retinopathy: clinical features and complement-mediated microvascular injury mechanisms

**DOI:** 10.3389/fimmu.2026.1847573

**Published:** 2026-07-09

**Authors:** Igor Resnick, Mladena Radeva

**Affiliations:** 1 Stem Cell Biology Laboratory, Research Institute, Medical University of Varna, Varna, Bulgaria; 2 Specialised Eye Hospital - Varna, Medical University of Varna, Varna, Bulgaria

**Keywords:** CAR-T cell therapy, complement activation, hematopoietic stem cell transplantation (HSCT), Purtscher-like retinopathy, transplant-associated thrombotic microangiopathy (TA-TMA)

The recent work by Li et al. (1) presents a rare and clinically complex case of the development of Purtscher-like retinopathy (PLR) in an 11-year-old boy with B-cell acute lymphoblastic leukemia. According to the publication, the patient received a CODPL regimen followed by methotrexate, vindesine, and idarubicin (22 consolidation cycles). Two and a half years later, the boy developed a full-blown relapse treated with CD19- and CD22-targeted CAR-T cells. The second relapse, with extramedullary (non-CNS) involvement, occurred 7 years after diagnosis. Treatment with inotuzumab ozogamicin followed by belimumab led to molecular remission, after which allogeneic hematopoietic stem cell transplantation (HSCT) from a 10/10 matched unrelated donor was performed using myeloablative TBI-containing (10 Gy) conditioning. In the post-transplant period, viral infections were noted (ADV, HHV-6, and H1N1). Against this background, on day +160, PLR developed. It significantly improved after injections of a drug referred to as “lezumab” in both eyes. On day +194, transplant-associated thrombotic microangiopathy (TA-TMA) was diagnosed. Throughout the post-transplant period, the patient demonstrated full-donor chimerism. The presented case is well documented and clinically complex. At the same time, the interpretation proposed by the authors raises questions. The pathophysiology of PLR is currently still not well defined. The condition was described more than a 100 years ago; however, a unified concept of its development is lacking (2, 3). It is considered an ischemic injury of the retinal nerve fiber layer due to occlusion of peripapillary terminal arterioles caused by various factors, such as thrombosis or microembolization by fat emboli and leukocyte aggregation induced by different factors. PLR has been associated with numerous conditions, notably trauma (complement activation), acute pancreatitis (systemic release of proteases and lipase), and systemic lupus erythematosus (immune complexes), as well as numerous other disorders. They are often regarded as related comorbidities rather than a direct relationship, with at most a potential causal link, as the putative “triggers” are heterogeneous (4, 5).

In the present case, there are multiple factors previously associated with the development of PLR in the post-transplant setting. These include intensive multi-agent chemotherapy, radiation therapy, HSCT, immunotherapy, immunosuppressive treatment, and viral infections. Under such conditions, it is difficult to identify a single causal factor, especially in the absence of a unified etiology, a well-defined pathogenetic mechanism, and the presence of only clinical diagnostic criteria.

As indicated by the title, the primary focus of this article is CAR-T cell therapy. CAR-T cells were administered approximately 3 years prior to the onset of ophthalmologic symptoms. After subsequent allo-HSCT, complete donor chimerism in blood and bone marrow was achieved. Li et al. provided no evidence for persistence of CAR-T cells at the time of PLR onset, as no CAR-T-specific monitoring data were reported. Thus, there is no evidence of a cellular substrate for the hypothesized interaction. Therefore, the available evidence does not sufficiently support an association between prior CAR-T cell therapy and the development of PLR.

At the same time, the authors estimated another mechanism of PLR development, in which a key role is assigned to complement activation. Complement may contribute to endothelial injury in PLR (6–8) but is not a direct mediator of CAR-T cytotoxicity (9), and its rise during cytokine release syndrome (absent in this patient) is limited to the early post-infusion period (10). In the presented case, PLR was diagnosed approximately 3 years after two administrations of CD19- and CD22-directed CAR-T cells. At the onset of PLR, aqueous humor analysis revealed elevated levels of bFGF and VCAM-1, findings interpreted by Li et al. as evidence of blood–retinal barrier disruption. However, no measurements of complement activation markers were reported in either ocular fluid or peripheral blood at the time of PLR onset, during subsequent involvement of the fellow eye, or throughout the period of treatment response and clinical recovery.

The only reported measurement of the terminal complement complex C5b-9 (298 ng/mL) was obtained in peripheral blood 34 days after PLR onset. This assessment was performed in the setting of clinically apparent TA-TMA, characterized by thrombocytopenia, proteinuria, renal dysfunction, and other manifestations of systemic endothelial injury. No serial measurements of complement activation markers or other complement components were reported.

TA-TMA is characterized by endothelial injury, microvascular dysfunction, and thrombosis of small vessels. The retina is part of the microvascular bed and is susceptible to ischemic injury, similar to the kidneys, brain, lungs, and gastrointestinal tract. In this context, the ophthalmologic findings warrant consideration within the broader spectrum of microangiopathic processes. Ocular involvement interpreted as PLR has been reported in primary thrombotic microangiopathies (11) and, although uncommon, also in association with TA-TMA. Notably, previously reported cases usually share several clinical features with the present patient, including young age, acute lymphoblastic leukemia or lymphoma, exposure to intensive multi-agent chemotherapy, HSCT, myeloablative conditioning, total body irradiation, and infectious complications (12–15). Nevertheless, the available evidence remains limited, and the relationship between PLR and subsequent TA-TMA in the present case cannot be established with certainty.

Therefore, the available data do not permit assessment of whether complement activation contributed to the development of PLR in this case and do not provide sufficient evidence to support complement activation as a leading mechanism underlying the retinal manifestations observed.

A separate comment concerns the role of the TTC37 variant. Biallelic loss-of-function mutations in TTC37 cause autosomal recessive trichohepatoenteric syndrome (THES1, OMIM #222470) (16) often associated with antibody deficiency. In the present report, the patient was described as carrying a class II TTC37 variant associated predominantly with antibody deficiencies. No further information regarding the specific variant was provided. To date, no established association has been reported between heterozygous TTC37 variants and endothelial injury, complement activation, Purtscher-like retinopathy, transplant-associated thrombotic microangiopathy, or HSCT outcomes. Therefore, the relevance of this finding to the clinical manifestations described in the present case remains uncertain.

The manuscript also contains an important factual inconsistency regarding the reported immunotherapy. Throughout the article, including Table 2, the authors refer to belimumab, which is a BAFF (BLyS)-targeting monoclonal antibody used primarily in autoimmune diseases (17). However, in the Discussion, this agent is described as “a bispecific T-cell engager that binds CD3 on T cells and CD19 on B cells, activating T cells to lyse tumor cells.” This description is incompatible with belimumab but is fully consistent with blinatumomab, a CD19/CD3-bispecific antibody used in the treatment of B-cell acute lymphoblastic leukemia (18). Furthermore, the reference cited by Li et al. in this context concerns blinatumomab rather than belimumab. Therefore, the original report appears to contain a drug-name error that should be corrected. Clarification of this point is important because the discussion of immune activation and endothelial interactions in the manuscript is based on the biological properties of blinatumomab rather than belimumab. To avoid confusion among readers, correction of this apparent error would be highly desirable.

The presented case is of interest because it combines a rare ophthalmologic syndrome with multiple complications associated with ALL treatment. At the same time, its interpretation as a consequence of a CAR-T-triggered complement-mediated immune mechanism is not sufficiently supported by the available data. A more cautious interpretation, taking into account multifactorial influences and the possible role of microangiopathy, appears justified.
